# Review of Computational Fluid Dynamics Analysis in Biomimetic Applications for Underwater Vehicles

**DOI:** 10.3390/biomimetics9020079

**Published:** 2024-01-28

**Authors:** Zhijun Zhang, Qigan Wang, Shujun Zhang

**Affiliations:** 1 Key Laboratory of CNC Equipment Reliability (Ministry of Education), School of Mechanical and Aerospace Engineering, Jilin University, Changchun 130022, China; wangqg21@mails.jlu.edu.cn; 2 School of Computing and Engineering, Gloucestershire University, Cheltenham GL50 2HR, UK

**Keywords:** underwater vehicles, bioinspired design, biomimetic propulsion, drag reduction, noise reduction, computational fluid dynamics (CFD)

## Abstract

Biomimetics, which draws inspiration from nature, has emerged as a key approach in the development of underwater vehicles. The integration of this approach with computational fluid dynamics (CFD) has further propelled research in this field. CFD, as an effective tool for dynamic analysis, contributes significantly to understanding and resolving complex fluid dynamic problems in underwater vehicles. Biomimetics seeks to harness innovative inspiration from the biological world. Through the imitation of the structure, behavior, and functions of organisms, biomimetics enables the creation of efficient and unique designs. These designs are aimed at enhancing the speed, reliability, and maneuverability of underwater vehicles, as well as reducing drag and noise. CFD technology, which is capable of precisely predicting and simulating fluid flow behaviors, plays a crucial role in optimizing the structural design of underwater vehicles, thereby significantly enhancing their hydrodynamic and kinematic performances. Combining biomimetics and CFD technology introduces a novel approach to underwater vehicle design and unveils broad prospects for research in natural science and engineering applications. Consequently, this paper aims to review the application of CFD technology in the biomimicry of underwater vehicles, with a primary focus on biomimetic propulsion, biomimetic drag reduction, and biomimetic noise reduction. Additionally, it explores the challenges faced in this field and anticipates future advancements.

## 1. Introduction

Biomimetics and bio-inspiration, distinct yet complementary, both derive insights from nature’s ingenuity in science and engineering. Biomimetics, specifically for underwater vehicles, studies and emulates the efficient shapes and behaviors of aquatic creatures like fishes [1,2,3], dolphins [4,5,6], and whales [7,8,9], leading to innovative underwater vehicle designs with enhanced speed, thrust, maneuverability, and reduced water drag. Meanwhile, bio-inspiration, adopting a broader perspective, seeks abstract inspiration from nature, influencing diverse fields. It applies nature’s principles to foster innovation, extending beyond the direct replication of natural systems.

In this article, we intertwine biomimetics and bio-inspiration, portraying them as part of a unified narrative. They collectively emphasize nature’s role in inspiring practical, technological solutions. Table 1 presents the Reynolds number ranges for various aquatic organisms, alongside those of typical Autonomous Underwater Vehicle (AUV) models. This comparison sets the stage for our in-depth discussion on biomimetics. The diversity in Reynolds number ranges shown in the table reflects the broad spectrum of adaptations and efficiencies found in aquatic life. This diversity acts as a rich source of inspiration for the design and development of AUVs, embodying the core principles of biomimetic and bio-inspired design.

CFD is pivotal in the development of underwater vehicles, offering predictions and simulations of fluid behavior. This technology aids designers in optimizing underwater vehicle structures at early design stages, enhancing efficiency and resource conservation. Moreover, CFD has demonstrated close alignment with experimental outcomes, positioning it as an efficient alternative for underwater vehicle experiments. A prominent example of this is the SUBOFF model [10]. Developed by the United States Naval Surface Warfare Center Carderock Division (NSWCCD), the SUBOFF model encompasses both fully appended and bare hull forms. It was specifically designed for experimental studies and CFD research. As a benchmark model, it facilitates the study of hydrodynamic characteristics in submarine-like bodies, focusing particularly on aspects like drag, flow separation, boundary layer transitions, and vortex generation [11].

**Table 1:** The Reynolds number ranges of various aquatic organisms and typical AUVs.

Biological Species	Reynolds Number	Reference	AUV Models	Reynolds Number	Reference
American eel	1006	[12]	Pirajuba	3.5 × 10^6^	[13]
Bluegill sunfish	1440	[14]	Spray	4 × 10^5^–6 × 10^5^	[15]
Yellowfin tuna	1.9 × 10^4^	[12]	Slocum	5.2 × 10^5^–7.5 × 10^5^	[16]
Cownose ray	10^5^	[17]	Seaglider	4.9 × 10^5^	[18]
Scomber scombrus	3.2 × 10^5^	[19]	SeaExplorer	4.9 × 10^5^–9.9 × 10^5^	[16]
Manta ray	5.1 × 10^5^	[20]	Petrel-II	7.2 × 10^5^–1.8 × 10^6^	[21]
Greenland shark	10^6^	[22]	Petrel-L	7.7 × 10^5^	[23]
longfin inshore squid	4.4 × 10^5^–1.5 × 10^6^	[24]	Petrel-4000	1.2 × 10^6^	[25]
Basking shark	6.9 × 10^6^	[26]	ALBAC	7 × 10^5^–1.4 × 10^6^	[27]
Humpback whale	8.8 × 10^6^	[7]	Miniature	5.5 × 10^4^	[28]
Dolphin	2.3 × 10^6^–1.6 × 10^7^	[29]	ALEX	9.9 × 10^4^–4.9 × 10^5^	[30]

Researchers and engineers utilize the model to validate CFD codes, reinforcing the reliability of CFD [31,32,33]. Additionally, the application of CFD in validating factors like propeller thrust [34,35,36] and hydrodynamic noise [37,38,39] further affirms its dependability and efficiency.

### 1.1. CFD Methodologies in Underwater Vehicle Design

Integrating CFD with biomimetics represents a pioneering approach in underwater vehicle design, promising novel solutions to complex challenges. This interdisciplinary approach holds the promise of enriching both natural science and engineering technology.

The analysis in CFD predominantly depends on solving the Navier–Stokes equations, nonlinear partial differential equations that describe fluid motion. In CFD, the computational domain is typically discretized into many small elements or control volumes. The flow equations within each element are solved, providing valuable information about fluid behavior.

Among the key approaches in CFD, the Reynolds-Averaged Navier–Stokes (RANS) method is notable for its application in simulating turbulent flows. Implemented often using numerical techniques like the Finite Volume Method (FVM), known for its conservational properties, RANS uses specific turbulence models, such as *k-ω* and *k-ε*, to enhance turbulence prediction. Additionally, the Unsteady Reynolds-Averaged Navier–Stokes (URANS) extends RANS to include time-dependent effects, enabling the simulation of unsteady flows. URANS balances computational efficiency with the ability to capture transient phenomena, often utilizing FVM for discretization.

Further advanced methods in CFD include Large Eddy Simulation (LES) and Detached Eddy Simulation (DES). LES directly resolves large-scale turbulent structures while modeling smaller scales, making it suitable for detailed turbulence studies and complex flows. DES, a hybrid approach, combines RANS in near-wall regions with LES away from walls. Both methods require finer grid resolution and are computationally more intensive than RANS. In addition, Direct Numerical Simulation (DNS) resolves all scales of turbulence directly, without modeling, providing the most detailed and accurate flow predictions. However, DNS is computationally very demanding and is primarily used for fundamental research.

Numerical methods also play a crucial role in CFD. The FVM is widely used for discretizing equations in CFD, especially in RANS and URANS simulations, due to its conservation properties and versatility. The Finite Element Method (FEM) is used for complex geometries and boundary conditions, effective in structural analysis and fluid dynamics, though less commonly used in turbulent flow simulations than FVM. The Lattice Boltzmann Method (LBM), a particle-based CFD technique, is notable for simulating complex boundaries, multiphase flow, and multicomponent flow issues. It stands out for its kinetic approach, differing from FVM and FEM. Lastly, the Immersed Boundary Method (IBM) is suitable for handling complex, moving, and elastic boundaries. Combined with LBM (LBM-IBM), it effectively addresses flow problems with complex geometries and moving boundaries, including Fluid–Structure Interactions (FSIs).

This diversity of methods and approaches in CFD allows for tailored solutions to specific problems, such as biomimetic designs for underwater vehicles, where the intricate interplay between fluid dynamics and structure can be accurately captured and studied. The evolution of these methods continues to enhance understanding and capabilities in designing efficient and effective underwater vehicles. Additionally, Table 2 provides a brief overview of CFD methods and numerical techniques for fluid dynamics simulations.

### 1.2. Review Framework

To comprehensively explore the application of CFD in the biomimetic field of underwater vehicles, this review follows the systematic literature search and screening framework outlined in the PRISMA guidelines. The primary databases searched were Web of Science and Scopus. The search timeframe was set from January 2000 to January 2024 to ensure coverage of the most recent research advancements. This review’s search strategy focuses on the application of CFD in diverse biomimetic designs of underwater vehicles, specifically targeting studies that imitate biological motion mechanisms, such as bio-inspired hydrofoil flapping propulsion, biomimetic fish caudal fin propulsion, batoid-style propulsion, dolphin-style propulsion, squid-style propulsion, and biomimetic technologies for drag and noise reduction. To accurately identify the relevant literature, we used a series of Boolean operator combinations in the titles, abstracts, and keywords of papers, including (Underwater OR Vehicle OR “Underwater Robotics” OR AUV OR Hydrofoil) AND (“Computational Fluid Dynamics” OR CFD) AND (bio-inspired OR biomimetics OR biomimicry). This strategy aims to identify articles that extensively explore CFD in these specific biomimetic applications.

The focus was on academic papers published in English to guarantee the accuracy and quality of the information. The purpose of this review is to systematically evaluate and summarize the current applications and development trends in CFD in the biomimetic design of underwater vehicles.

In addition to systematic literature searches in the Web of Science and Scopus databases, we consulted Google Scholar to supplement and corroborate the information. This approach was primarily used to supplement the literature in the Introduction and other sections, such as gathering comparative information on the trailing-edge serrated structures in aviation and maritime applications.

**Figure 1:**
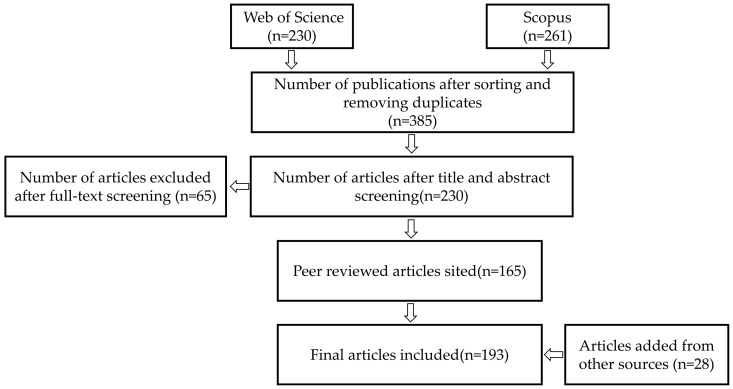
Flow chart of the literature source selection process.

Figure 1 presents a flow chart that outlines the literature selection process for this article. In this process, the literature was initially screened from the Web of Science and Scopus databases by title, resulting in the exclusion of 106 duplicates.

We subsequently reviewed the titles and abstracts, eliminating 155 articles unrelated to underwater vehicles, primarily those focusing on aircraft research. In the next stage of filtering, a comprehensive review of the full texts was performed.

Articles without content on hydrofoil-like caudal fin flapping propulsion, biomimetic robotic fish propulsion, biomimetic batoid-style, dolphin-style, and squid-style propulsion, and biomimetic drag and noise reduction methods were specifically excluded. Additionally, articles lacking adequate CFD research content were also excluded.

Our focus in the field of robotic fish propulsion research included articles involving FSI models, caudal fin propulsion mechanisms in robotic fish, a detailed analysis of wake structures, and studies on the maneuverability and flexibility of robotic fish in diverse aquatic environments. Additionally, special attention was given to research that applied data-driven methods and multidisciplinary, multi-objective optimization strategies in the design of robotic fish.

Ultimately, these carefully selected articles were supplemented with additional literature from Google Scholar to compile this article’s reference section.

This article’s structure is organized as follows: Section 2 introduces biomimetic propulsion, with Section 2.1 focusing on hydrofoil-like caudal fin flapping propulsion, Section 2.2 covering robotic fish propulsion, Section 2.3 discussing batoid-like propulsion, Section 2.4 discussing dolphin-style propulsion, and Section 2.5 discussing squid-style propulsion. Section 3 focuses on biomimetic drag reduction, and Section 4 explores biomimetic noise reduction. Section 5 addresses challenges and limitations in applying CFD to biomimetic underwater vehicle applications and offers perspectives on future developments. The final section, Section 6, provides a summary of this article’s content.

## 2. Applications of Biomimetic Propulsion

AUVs have garnered significant interest due to their extensive applicability and multifunctional utility. These applications include but are not limited to, deep-sea exploration [40,41], seabed geological research [42,43], marine resource extraction [44], and underwater infrastructure maintenance [45,46]. However, the conventional propulsion method of AUVs, relying on propellers, poses several significant challenges. These challenges encompass excessive energy consumption, increased resistance, and heightened noise pollution, especially in complex marine environments.

Consequently, scientists have been observing and analyzing the swimming and maneuvering techniques of aquatic creatures, dedicating themselves to the development of innovative, biomimetic AUVs. These organisms use their physical structures and aquatic maneuverability for propulsion, producing undulating movements through the coordination of body, pectoral fins [47], and tail movements [48,49,50]. They also manipulate the surrounding water flow. This innovative approach offers a propulsion system that not only surpasses the speed and noise reduction capabilities of traditional AUVs but also draws attention to its groundbreaking energy efficiency and maneuverability.

This section provides a focused review of the application of CFD technology in the field of biomimetic propulsion for underwater vehicles.

### 2.1. Biomimetic Hydrofoil-like Tail Fin Propulsion

Inspired by aquatic organisms, the propulsion technique utilizing tail-fin flapping offers significant benefits, including enhanced propulsion efficiency, increased maneuverability and flexibility, and the ability to maintain stability in complex environments. These advantages unlock vast potential for the design of highly efficient and adaptable underwater vehicles.

**Figure 2:**
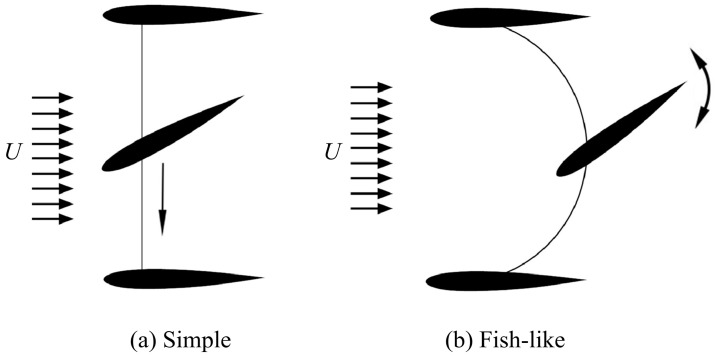
Schematic of flapping hydrofoils. (**a**) Simple motion pattern, and (**b**) fish-like motion pattern. Reproduced with permission from [54].

**Figure 3:**
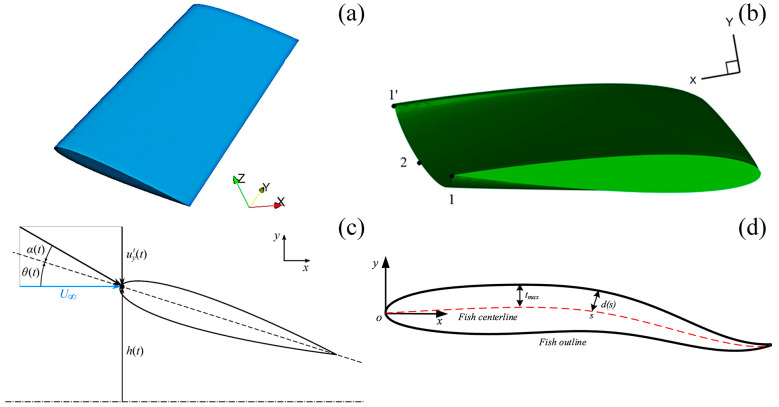
Different types of 3D and 2D hydrofoils. (**a**) The 3D NACA0012 flapping foil, reproduced with permission from [55]. (**b**) The 3D flexible NACA foil, reproduced from [63], CC BY 4.0. (**c**) The NACA0015 foil, reproduced from [61], CC BY 4.0. (**d**) The fish-like NACA0012 foil, reproduced with permission from [58].

Numerous researchers have performed numerical simulations on biomimetic propulsion using caudal fin-like hydrofoils [51,52,53]. They have endeavored to uncover the fundamental relationship between design parameters such as frequency, amplitude, aspect ratio, and others, and their effects on propulsion efficiency and other dynamic performances. Figure 2 illustrates schematic diagrams of two typical flapping hydrofoil motion patterns. In Figure 2a, the foil begins the down-stroke phase in a straight direction normal to free stream, *U*. In Figure 2b, the foil moves along the circular direction during the down stroke. This circular path introduces an additional velocity component to the flapping system and alters the kinematics of the foil’s motion, thereby influencing the propulsive performance of the flapping foil [54]. Furthermore, Table 3 offers a concise summary of various hydrofoils, detailing their structures, dimensions, Reynolds numbers, and numerical simulation methods used. Among these, some different three-dimensional (3D) and two-dimensional (2D) hydrofoils are illustrated in Figure 3.

**Table 3:** Summary of numerical simulation of flapping in hydrofoil-like structures.

Type of Hydrofoil	Dimensional	Reynolds Number	Numerical Method	Reference
NACA0012	3D	100–600	LBM-IBM	[55]
NACA0012	2D and 3D	4.4 × 10^6^	*k-ω*	[56]
NACA0005	2D	5 × 10^2^–5 × 10^4^	SST	[57]
NACA0012	2D	45,000	URANS	[58]
NACA0012	2D	307,000	*k-ω*	[59]
NACA0012	2D	40,000	*k-ω*	[60]
NACA0015	2D	300,000	URANS	[61]
NACA0012	2D	10^5^–8 × 10^5^	*k-ω*	[62]
NACA0013	3D	11,000	URANS	[63]
NACA0012	2D	42,000	URANS	[64]
NACA0012	2D	40,000	URANS	[65]
NACA0012	3D	200	LBM-IBM	[66]
NACA0012	3D	50,000	*k-ε*	[67]
NACA0012	2D	400	LBM-IBM	[68]
NACA0012	2D	1500	IBM	[69]
NACA0012	2D	5000	IBM	[70]
NACA0012	2D	5000	IBM	[71]
NACA0012	2D	500	LBM-IBM	[72]
NACA0012	2D	195,000	SST	[73]
NACA0012	2D	2000	DSD/SST	[74]
NACA0012	2D	4000	IBM	[75]
NACA0012	2D	20,000	URANS	[76]
NACA0012	2D	9000–13,600	IBM	[77]
NACA0015	2D	260,000	URANS	[78]
NACA0012	2D	500–5000	BEM	[79]
NACA0015	2D	3000	FVM	[80]
NACA0012	2D	42,000	*k-ε*	[81]
NACA0012	2D	40,000	*k-ω*	[82]

In the study of flapping-hydrofoil propulsion mechanisms at low Reynolds numbers, a significant number of research efforts have been directed toward understanding the impact of the hydrofoil’s shape and motion patterns on propulsion efficiency.

For instance, Karbasian et al. [54] and Gupta et al. [83] explored the influence of hydrofoil shape on propulsive performance. Karbasian et al. [54], drawing inspiration from fish fin morphology, introduced a fish-like flapping hydrofoil motion pattern. In contrast, Gupta et al. [83] focused on how different hydrofoil shapes affect the strength of wake vortices.

Additionally, Abbaspour and Ebrahimi [84], as well as Han et al. [55], compared the propulsive characteristics of hydrofoils under flapping and oscillating mechanisms and examined the impact of viscosity on flapping hydrofoil performance.

Abbaspour and Ebrahimi [84] observed pronounced leading-edge vortices in the wakes of flapping hydrofoils, while Han et al. [55] utilized the LBM-IBM method to investigate the flow field characteristics of 3D flapping hydrofoils across various Reynolds numbers. Moreover, certain studies have concentrated on hydrofoil flexibility and motion modes. You et al. [85] created deformable hydrofoils that mimic fish or cetacean fin propulsion, while Martin et al. [56] and Wei et al. [58] investigated the effects of varying Strouhal numbers and wavelengths on the propulsive performance of a NACA0012 hydrofoil.

Furthermore, Vijayakumaran and Krishnankutty [60], along with Alberti et al. [61], scrutinized the effects of diverse motion parameters, including Strouhal number, angle of attack, pitch amplitude, and phase angle, on hydrofoil propulsion.

Vijayakumaran and Krishnankutty [60] explored hydrofoils that combine swinging and yawing movements, whereas Alberti et al. [61] focused on NACA0015 hydrofoils performing combined sinusoidal rise and pitch motions.

To advance this field further, Liu et al. [62] and Zhou et al. [63] utilized CFD methods to study self-propelled NACA0012 hydrofoil models and a biomimetic NACA0013 hydrofoil, while analyzing various factors affecting their propulsive performance.

Finally, Zhang et al. [65] and Khalid et al. [71] introduced innovative hydrofoil design and motion paths. Zhang et al. [65] proposed a flapping hydrofoil with a three-degree-of-freedom motion path, and Khalid et al. [71] studied the fluid dynamics performance of NACA0012-like hydrofoils at different Reynolds numbers using an IBM-based computational solver, investigating the effects of wavelength and Strouhal number as control parameters.

These studies collectively indicate that the efficiency of hydrofoil propulsion and its fluid dynamics performance are multifaceted issues, involving factors such as hydrofoil shape, motion patterns, and the fluid environment. For example, comparisons between flapping and oscillating mechanisms reveal differences in vortex structures and propulsion efficiency across varying motion patterns.

Meanwhile, studying the effects of different motion parameters, such as Strouhal number, angle of attack, and pitch amplitude, unveils the complexity involved in designing more efficient hydrofoil systems.

Additionally, by mimicking biological motion characteristics and introducing new paths of motion, such as multi-degree-of-freedom paths, researchers are exploring novel ways to enhance hydrofoil propulsion efficiency.

These studies not only complement each other, providing a more comprehensive understanding of hydrofoil propulsion mechanisms but also lay the theoretical and experimental groundwork for designing future efficient propulsion systems.

### 2.2. Biomimetic Robotic Fish Propulsion

The exceptional propulsion performance and agile maneuverability of fish undoubtedly arouse interest in biomimetic robotic fish research. These biomimetic robotic fish play a crucial role in the design and analysis of underwater propulsion devices, prompting academia to intensify research and discussion on this subject [2,86,87,88,89]. Particularly for the application in small AUVs, biomimetic underwater robotic fish propulsion devices hold immense potential and could be extensively exploited and utilized in future scientific research.

It is widely recognized that the swimming method of fish will significantly influence the design of future robotic fish. The oscillatory motion of the fish’s tail and abdomen significantly affects the surrounding fluid flow. However, due to the instability in these effects, a comprehensive understanding and analysis of vortex dynamics and FSI are required.

In this process, CFD numerical simulations have made significant contributions to the research of many scholars. Numerous researchers have investigated the hydrodynamic performance of robotic fish using this method [3,90,91,92], advancing future related research. Lamas and Rodriguez [93] conducted a comprehensive review of numerical simulations in hydrodynamics and biomimetic propulsion, highlighting the importance of numerical simulations in studying fish swimming patterns.

**Figure 4:**
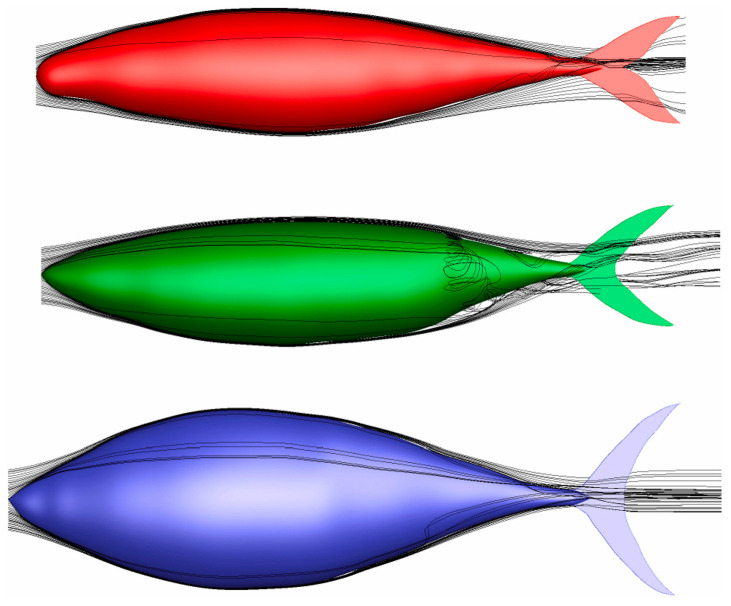
Three-dimensional streamlines around three species of fish at a Re of 318,000. Reproduced with permission from [19].

To address the scarcity of reference geometric models for freshwater fish and the inadequacy of applicable numerical methods, Khan et al. [94] developed a numerical model utilizing OpenFOAM. This model used a realistic fish-shaped geometric model and was calibrated with laboratory-measured values. Similarly, Düzbastilar and Şentürk [19] developed Computer-Aided Design (CAD) models for three fish species (Scomber scombrus, Sarda sarda, and Thunnus thynnus) and conducted numerical simulations to assess their drag and propulsion performance. Figure 4 illustrates the 3D streamlines corresponding to the three fish species at a Reynolds number of 318,000.

Regarding the application of FSI models, Fouladi and Coughlin [95] proposed an FSI model to simulate the swimming behavior of fish in water. This model, utilizing commercial CFD software and user-defined functions, facilitates establishing numerical simulations of oscillatory fish swimming behaviors and serves as a reference for developing hydrodynamic numerical models for biomimetic underwater vehicles. Chung et al. [96] used an FSI computational framework using accurate Riemann solvers and the FVM to simulate the flapping behavior of fish fins and joint systems. The coupling of CFD and Computational Structural Dynamics (CSD) solvers enabled them to examine the impact of bidirectional FSI on fluid flow, and they corroborated their findings with experimental data.

Furthermore, Wright et al. [97] utilized FSI analysis to investigate how the material properties of robotic fish caudal fins affect hydrodynamic performance and efficiency. In a distinctive approach, Li et al. [98] studied live pufferfish and developed a numerical model, integrating CFD with multibody dynamics. Their study focused on fluid–fish interactions and highlighted the influence of flexible fins on the propulsion performance of fish.

Following this, by combining biomimetic design with fluid dynamics, Zangeneh and Musa [99] simulated the swimming of fish in water using OpenFOAM’s dynamic mesh technology to investigate their hydrodynamic characteristics. This research not only enhanced the understanding of underwater robotic fish’s dynamic behaviors but also provided valuable insights for designing, remotely controlling, and optimizing their flexibility. Collectively, these studies underscore the importance of using diverse methods and perspectives in understanding and optimizing the hydrodynamic characteristics of underwater vehicles.

Also focusing on caudal fin propulsion, Palit et al. [100] conducted a detailed CFD analysis on tilapia, focusing their research on how tail and abdominal vibrations influence tilapia’s hydrodynamic characteristics. They particularly emphasized variations in drag and lift coefficients, providing crucial insights into the dynamics of fish swimming. Their study complements the work of Chowdhury et al. [101], who constructed a robotic fish model that imitates the tail-fin propulsion mechanism of fish in order to assess its hydrodynamic properties during linear motion. Their findings provide a deep understanding of the role of caudal fin-driven mechanisms in enhancing the performance of biomimetic robotic fish, thus furthering advancements in robotic fish design.

Vignesh et al. [102] used CFD for both steady and unsteady simulations of bio-inspired AUVs, aiming to accurately calculate their hydrodynamic derivatives. Their research endeavors to provide key data for the design of more efficient AUVs. Meanwhile, Li et al. [103] used numerical simulations, focusing on the hydrodynamic performance of autonomously propelled tuna, including aspects like velocity, power requirements, and wake vortices. Their work offers valuable insights into the performance and driving mechanisms behind autonomous swimming, especially in terms of efficient propulsion and fluid dynamic optimization.

In their study of fish schooling behavior, Li et al. [104] investigated both the hydrodynamic characteristics and flow field structures of fish schools across various vertical modes, aiming to enhance the swimming efficiency of robotic fish schools. Pan and Dong [105], along with Ren et al. [92], conducted numerical simulations on fish in high-density, diamond-shaped schools to analyze the hydrodynamic interactions within the school. They discovered that fish in dense schools exhibit both higher thrust and improved propulsive efficiency compared with those in sparse schools, primarily due to the pronounced wall effect.

In the realm of motion control and design optimization of robotic fish, Tian et al. [106] developed a CFD simulation platform that focuses on adjusting the motion control parameters of robotic fish, thus offering new perspectives for design and optimization. Furthermore, the research by Ji et al. [107] and Zou et al. [108] focused on the practical functionalities of robotic fish, including object detection, tracking, and collision avoidance, thereby enhancing the performance and safety of robotic fish with CFD analysis. At the same time, Zhang et al. [109] and Chen et al. [110] used a comprehensive approach that combines data-driven methods and CFD technology. They developed multi-objective, multidisciplinary design optimization strategies for the motion control of biomimetic robotic fish, demonstrating the potential of technological integration in advancing robotic fish design.

**Figure 5:**
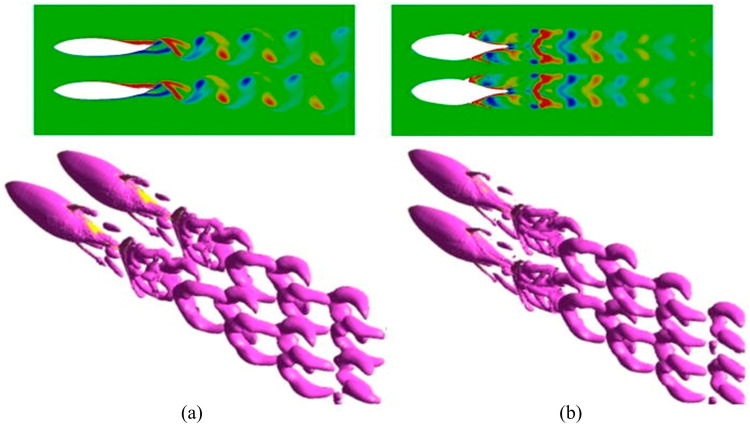
Vorticity contour in the mid-plane and the 3D wake structure visualized by the Q-criterion: (**a**) horizontal parallel pattern and (**b**) vertical parallel pattern. Reproduced with permission from [104].

Overall, CFD numerical simulations have played a crucial role in exploring the hydrodynamic performance of robotic fish and biomimetic underwater vehicles. From the realistic fish-shaped geometric models developed using OpenFOAM by Khan et al. [94] to the FSI analysis of robotic fish caudal fin material properties by Wright et al. [97], and the study of fish schooling behavior’s hydrodynamics conducted by Li et al. [104] (as shown in Figure 5), each study significantly contributes to enhancing our understanding of the hydrodynamics of underwater vehicles.

Moreover, these studies also emphasize the immense potential for technological integration. Examples including the CFD simulation platform by Tian et al. [106] and the multi-objective design optimization strategy by Chen et al. [110] highlight the importance of interdisciplinary collaboration in solving complex engineering challenges. With these advanced research efforts, researchers are not only able to design more efficient and flexible biomimetic robotic fishes but also gain a deeper understanding of the locomotion mechanisms of underwater organisms, thus profoundly impacting fields like marine engineering, environmental conservation, and biological studies.

### 2.3. Biomimetic Batoid-like Propulsion

Among aquatic organisms, batoids and rays use a distinctive swimming technique by flapping their pectoral fins, thereby exhibiting efficiency levels comparable to other fish species. Despite this, their superior agility and precision in executing turns distinguish them. Their streamlined bodies and low-drag skin contribute partially to this advantage. Their unique fin movement, facilitating greater propulsion with less energy, is equally critical. To replicate this biological characteristic, wherein batoids achieve maneuverability through pectoral fin flapping, researchers utilize CFD to perform detailed numerical simulations [111,112].

In the realm of simulating and understanding the dynamics of batoids, the work of Huang et al. [113,114] revealed how motion frequency, amplitude, and thrust interrelate in deformable airfoils inspired by batoids, delving into the hydrodynamic performance and wake structure of both the airfoil and Rhinoptera javanica. Similarly, studies by Bao et al. [115] and Luo et al. [116] uncovered dynamic pressure and velocity variations in the flapping fin motion of batoids, as well as the impact of pectoral fin movements on torque generation.

**Figure 6:**
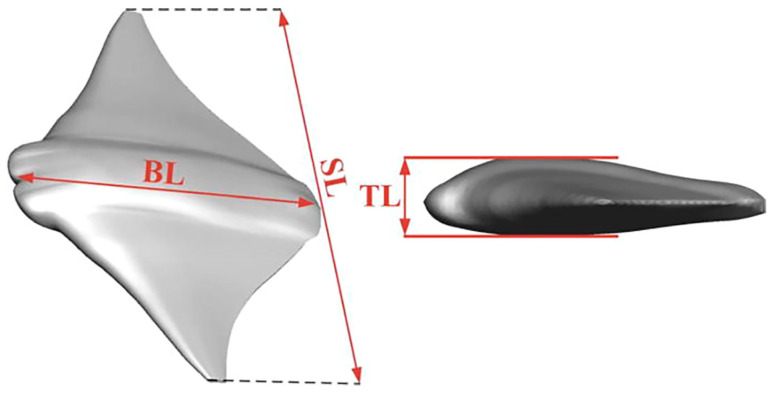
The manta ray model. Reproduced with permission from [117].

**Figure 7:**
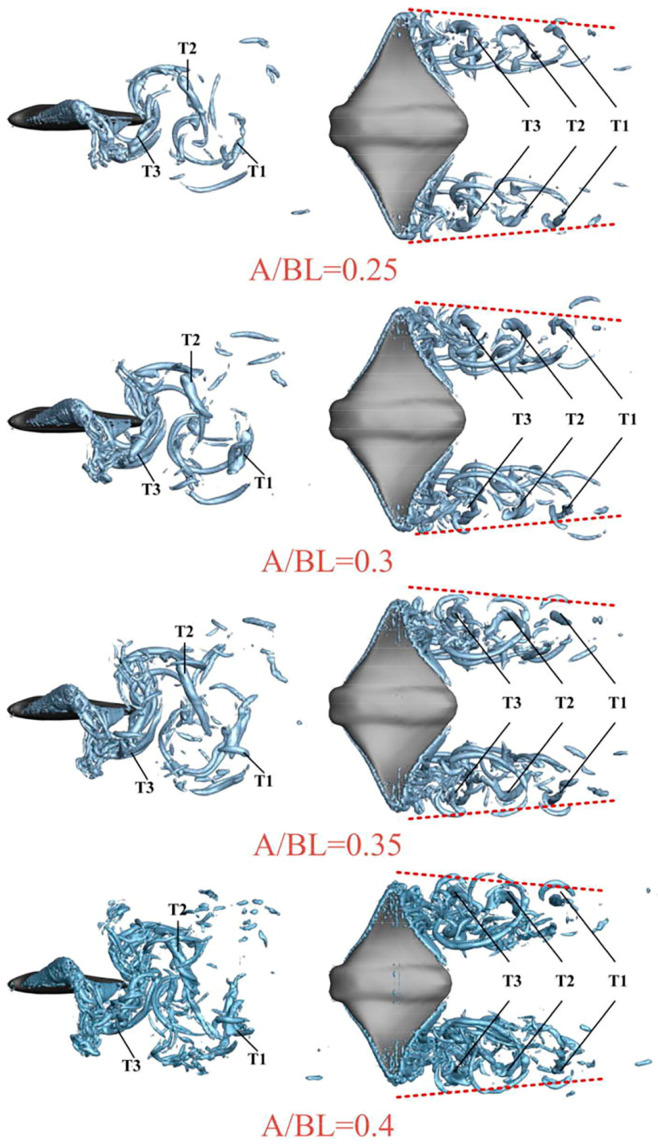
Three-dimensional wake structure at different amplitudes. Reproduced with permission from [117].

**Figure 8:**
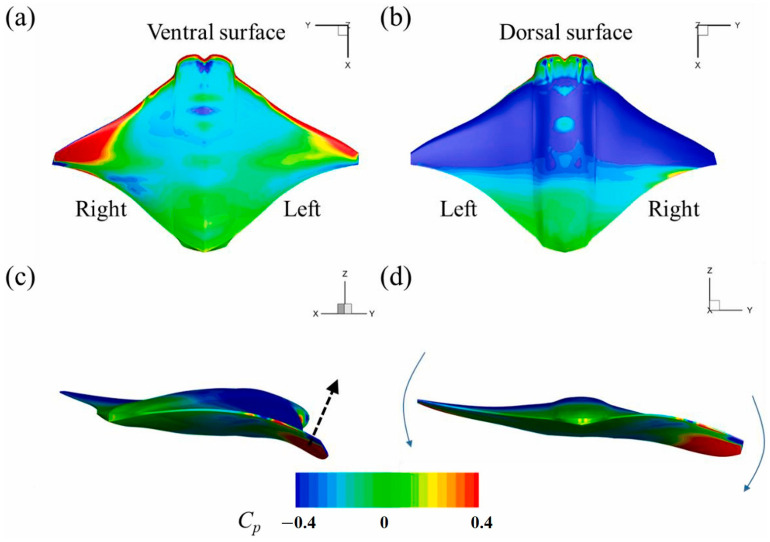
The pressure distribution on the ray’s body: (**a**) the pressure distribution on the ventral surface; (**b**) the pressure distribution on the dorsal surface; (**c**) the left and right fins fold in the horizontal plane; and (**d**) a component that forms a torque. Reproduced with permission from [116].

Regarding group swimming behaviors and their hydrodynamic effects, Gao et al. [117] conducted an in-depth investigation into the collective swimming behaviors of batoids and tuna, yielding new insights into the hydrodynamic effects of individual and coordinated swimming behaviors. The model of the manta ray under investigation is depicted in Figure 6, while Figure 7 presents the wake structure, and Figure 8 shows the pressure distribution on the ray’s body. Additionally, Menzer et al. [118] and Safari et al. [119] expanded on the exploration of complex unsteady vortex structures generated by the flapping movements of batoids using numerical simulations.

In the context of biomimetic batoid modeling and mechanisms, Lee and Kwon [120] utilized the commercial software package ADINA to simulate the journey distance and speed of a ray, while Rayapureddi and Mitra [121] developed an IBM-FSI algorithm using OpenFOAM to address challenges associated with biologically inspired self-propelled batoid robotic devices in 3D hydrodynamic flow fields. Furthermore, Liu et al. [20] proposed a novel design for a remotely controlled soft material robotic batoid. Separately, Huang et al. [122] conducted a hydrodynamic analysis using a six-degree-of-freedom motion equation, resulting in a design featuring dual pectoral fins and an auxiliary power vertical thruster. Abbaspour et al. [123] designed wave gliders of two different geometric shapes, showcasing the advantages of manta ray gliders in stable energy absorption.

Lastly, research by Bianchi et al. [124,125] focused on efficient locomotion mechanisms in underwater manta ray designs. They replicated the movement of the cownose ray for dynamic numerical analysis and utilized CFD models to study the hydrodynamic characteristics of ray swimming, thereby investigating efficient propulsion mechanisms.

Collectively, these studies lay a crucial foundation for a deeper understanding and simulation of batoid dynamics. Through in-depth exploration of the dynamics and motion mechanisms of batoids, these research efforts yield invaluable insights for the design of more efficient and agile underwater robotic batoids.

### 2.4. Biomimetic Dolphin Propulsion

Dolphins, recognized as some of the most remarkable swimmers among aquatic mammals, have always been admired for their efficient cruising capabilities. This efficiency primarily relies on their unique dorsoventral propulsion mechanism, which is widely used by other aquatic mammals. To a large extent, this influences the methods researchers use when seeking to understand and mimic the swimming mechanism of dolphins, particularly the underlying mechanisms of dorsoventral propulsion. Based on this, researchers often attempt to imitate dolphins from a kinematic perspective [126,127], as it is believed to be the quickest path to designing high-performance underwater vehicles. Many modern underwater vehicles’ design philosophies and technical inspirations stem from research on the propulsion movements of dolphins. Researchers further found that the interaction and coordination between a dolphin’s body and its pectoral, dorsal, and caudal fins substantially affect their swimming efficiency, offering the dolphin extraordinary agility and highly efficient propulsion power. Therefore, further deepening the understanding and interpretation of the interaction mechanism between dolphins’ bodies and their various fins, and how to mimic this mechanism, will be crucial in enhancing underwater vehicle design and exploring the propulsion mechanism of dolphin swimming.

Recent studies in the design of dolphin-inspired robotic systems have made significant strides, especially in simulating the locomotion mechanisms of dolphins and enhancing the hydrodynamic performance of these robots. Xue et al. [128] investigated the C-turning, pitching, and flapping propulsion mechanisms of a dolphin robot in their work, successfully proposing an accurate and stable maneuverability model. This model is vital for understanding and simulating the complex motion characteristics of dolphins.

Furthermore, Cao et al. [129] enhanced the pitching performance of dolphin robots by developing an elliptical-trajectory pectoral fin oscillation model. This innovation not only improved the control precision of the robot but also opened up new possibilities for its application in varying aquatic environments.

Wu and his team [130,131,132] utilized a comprehensive approach in the design of bionic dolphin robots. By integrating the advantages of mechanical dolphins and underwater gliders, they achieved significant improvements in maneuverability, speed, and endurance. In particular, the innovative biomimetic dolphin-like underwater glider described in reference [133] combines the agility of mechanical dolphins with the long-range stability of underwater gliders, demonstrating the efficient design of underwater robots achievable by simulating dolphin motion characteristics.

In the biomimetic study of dolphin hydrodynamic characteristics, Wang et al. [134] combined experimental and numerical methods to examine thrust generation, wake structure, and surface pressure of dolphins at different swimming speeds. Their research revealed that the dolphin’s caudal fin maintains a highly effective attack angle throughout most of each stroke and observed a significant difference in flow and surface pressure between low-speed and high-speed swimming.

Han et al. [135] delved into the dynamic characteristics of the dorsoventral propulsion mechanism of dolphins. They developed a 3D model of dolphin swimming and used an incompressible CFD solver based on the IBM approach to investigate the hydrodynamics and wake structure of dolphin swimming. Wang et al. [136] used theoretical analysis and numerical methods to calculate the swimming speeds of dolphins, highlighting the powerful thrust and efficient propulsion resulting from fin movement in high-speed swimming. Xia et al. [137] improved the understanding of dolphin motion mechanisms with a comparative analysis of different swimming modes.

**Figure 9:**
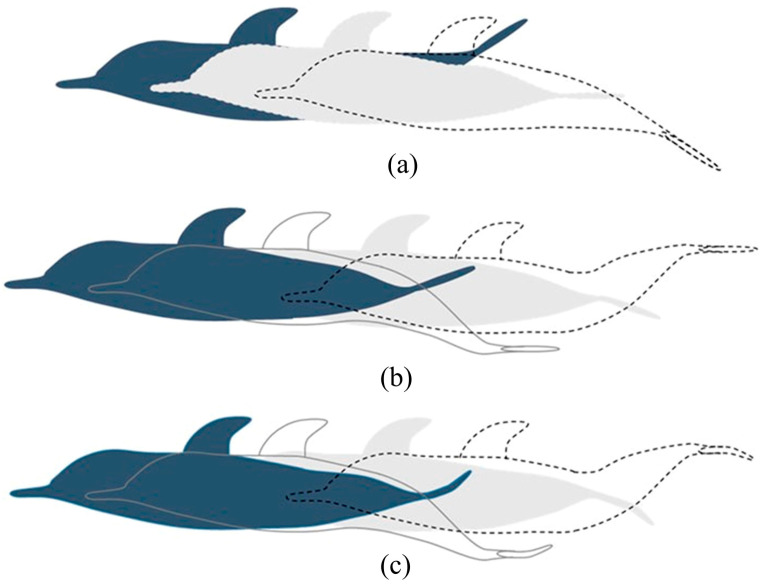
The postures of the three propulsion modes during dolphin movement: (**a**) single-stage propulsion; (**b**) double-stage propulsion; and (**c**) multi-stage propulsion. Reproduced from [29]. CC BY 4.0.

Tanaka et al. [138] recorded the swimming process of dolphins using high-speed camera technology and quantified the dynamics of dolphins during acceleration using CFD technology. Meanwhile, Feng et al. [29] divided dolphin motion into three phases: oscillation of the caudal fin, deformation of the caudal fin, and oscillation of the posterior third of the body, discussing the mechanisms for achieving fast and efficient propulsion. A schematic diagram of the postures of the three propulsion modes during dolphin movement is depicted in Figure 9.

**Figure 10:**
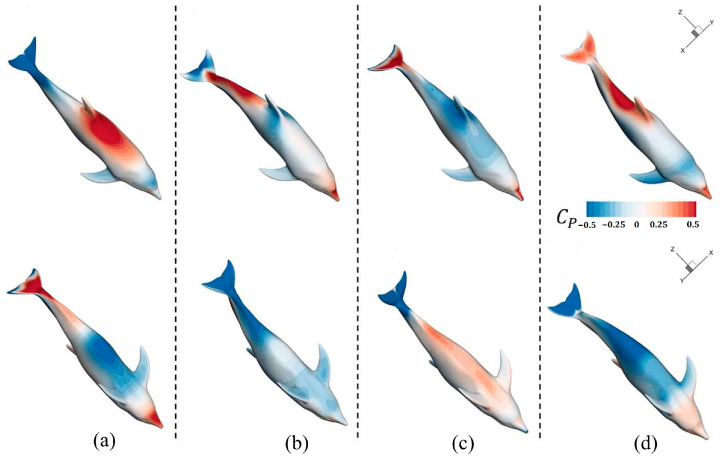
Surface contour of pressure coefficient on the oscillating dolphin body. (**a**) t/T = 0.25; (**b**) t/T = 0.50; (**c**) t/T = 0.75; (**d**) t/T = 1.00 of an oscillation cycle. Reproduced from [139]. CC BY 4.0.

Lastly, Guo et al. [139] developed a realistic 3D model of a dolphin and used CFD technology to investigate the oscillatory hydrodynamics of dolphins. Figure 10 depicts the surface contour of the pressure coefficient on the oscillating dolphin body, corresponding to a single cycle of dolphin movement. Their research emphasized the importance of oscillation and tail fin movement in generating strong thrust and effective lift, offering new perspectives for enhancing the hydrodynamic performance of underwater robots.

Collectively, these studies form an essential foundation for a deep understanding of dolphin hydrodynamic characteristics and CFD simulations, offering valuable references for designing efficient and agile underwater robots. The efficient swimming mechanisms of dolphins not only inspire the design of novel underwater robots but also pave the way for new directions and possibilities in the future development of underwater robotic technologies.

### 2.5. Biomimetic Squid Propulsion

The efficiency of marine organisms’ underwater movements is significantly enhanced by their streamlined external structures, which helps to reduce potential hydrodynamic drag. Furthermore, the propulsion methodology significantly influences acceleration duration and cruising speed during aquatic life’s movements. It is noteworthy that squid species exhibit a unique propulsion style, markedly distinct from that of other aquatic beings and the conventional propeller propulsion used by most underwater vessels.

Squids possess an extraordinarily effective jet propulsion mechanism [140,141,142]. This specialized evasion system is instantly activated in response to threats, particularly those posed by predators. This fast propulsion process provides sufficient thrust for squids to swiftly elude dangers, making their underwater evasion performance an effectively strategized survival mechanism.

Squids also exhibit superior underwater structural features, including streamlined body shapes that are highly hydrodynamic, designed to minimize the drag experienced during swimming. This combination of shape and hydrodynamics significantly reduces resistance during swimming, substantially enhancing the efficiency of their underwater movements.

The application of jet evasion provides squids with a significant advantage in acceleration. This unique acceleration capability, coupled with their remarkable hydrodynamic characteristics, enables squids to adeptly navigate a variety of complex oceanic conditions. This proficiency in underwater swimming not only highlights their exceptional skills but also attracts considerable scholarly interest for research in this area.

In the field of underwater soft robotics, the utilization of jet propulsion mechanisms of squids and other cephalopods represents a significant innovation. The study by Zhu and Xiao [143] offers a comprehensive overview of current research, highlighting the potential of jet propulsion technology in the development of soft underwater robots.

Olcay et al. [24] constructed a 3D squid model using tomography and computed the resistance, drag coefficient, swimming speed, and propulsion efficiency at varying nozzle diameters based on this model. Their findings highlight that the viscous resistance in squids with different Reynolds numbers is twice that of the pressure resistance and that expanding the nozzle diameter from 1 cm to 2 cm can lead to a 20% increase in propulsion efficiency. Their study elucidates the performance of jet propulsion mechanisms under varying physical parameters, which is essential for understanding and optimizing propulsion systems in underwater robots.

Additionally, they developed an improved squid model. The study revealed that the improved squid model requires minimal thrust during the acceleration phase of the time-dependent velocity profile [144]. This finding is informative for designing more efficient underwater propulsion systems. In another study, they discovered that using a larger nozzle diameter, smaller angles of attack, and eliminating fins could increase the propulsion efficiency of the squid to approximately 80%, thereby significantly enhancing propulsion efficiency [145].

Luo et al. [146] designed a 2D propulsion system to simulate squid swimming, inspired by the jet propulsion mechanism of squids and other cephalopods. Simulation analysis results indicate that higher Reynolds numbers result in a larger driving force and higher efficiency, which can be attributed to the strong jet-induced vortex and effective reduction in the external body vortex. In a turbulent environment, either increasing the Reynolds number or reducing the nozzle size will accelerate the formation of symmetry-breaking instability. These findings provide significant insights into simulating the performance of underwater robots in real marine environments.

In another study, they constructed a 3D pulsed jet propulsion model, composed of a flexible body and a controllable bending nozzle. The results validated the efficacy of curved nozzles for thrust vectoring and determined that the external surface viscous friction is predominantly influenced by variations in Reynolds numbers [147].

**Figure 11:**
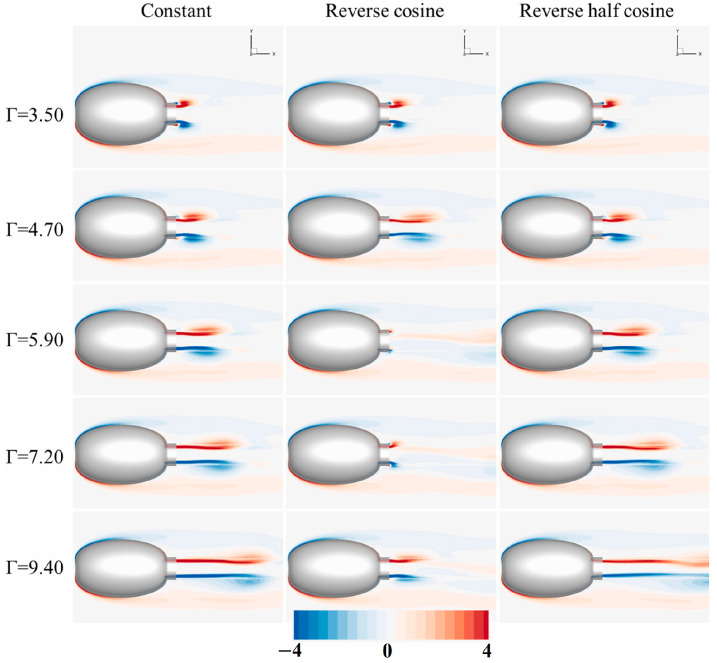
The evolution of the Z-vorticity distribution at plane z = 0 of different jet speed profiles. Reproduced with permission from [148].

Subsequently, they conducted a numerical study on a squid-inspired jet propulsion system by regulating body deflection during a single-emission process, aimed at investigating the impact of jet speed on the formation of a vortex ring and the system’s propulsion performance. The researchers concluded that at a specified maximum stroke ratio, the inverse-cosine jet speed can create a second vortex ring. The evolution of Z-vorticity distribution at plane z = 0 for various jet speed profiles is depicted in Figure 11. These findings offer valuable insights into the mechanical design and control of biomimetic jet propellers and underwater robots [148].

Hou et al. [149] simulated squids jumping out of the water using CFD technology. They analyzed the flow characteristics of squids in relation to the launch angle and carried out a quantitative analysis of the motion parameters of flying squids. The results demonstrated that jet propulsion tends to generate significant average thrust rather than high propulsion efficiency and revealed that the speed of flight is inversely related to the launch angle. These discoveries have contributed to performance enhancements in water-to-air transport tools.

Li and his team added tentacles to a robot squid in a simulation, analyzing the influence of the number, frequency, and maximum extension of these tentacles on propulsion capacity. Their findings suggested that a robot squid equipped with three tentacles achieves the best propulsion performance and that increasing the tentacle frequency can effectively enhance the steady-state velocity coefficient and propulsion efficiency. However, it is imperative to control the maximum bending range of the tentacles within a certain limit, as exceeding it may result in adverse effects [150].

In another study, they designed an underwater robot equipped with dual-driven composite tentacles, using overlapping grid technology to simulate incompressible viscous flow. After comparing three driving modes (reverse mode, homologous mode, and interlace mode), they discovered that the reverse mode demonstrated the best energy savings and propulsion efficiency. Compared with traditional fish-shaped robots, this underwater robot exhibited enhanced self-driving capabilities [151].

In summary, these studies provide rich insights into the understanding and optimization of underwater jet propulsion mechanisms, significantly contributing to the design and fabrication of more efficient, complex, and marine environment-adapted underwater robots and biomimetic propulsors.

## 3. Applications of Biomimetic Drag Reduction

During operation, underwater vehicles are subjected to drag forces induced by water flow. This can potentially reduce navigation speed, increase energy consumption, and affect both stability and operability. To mitigate these issues, biomimetic drag reduction technology, which emulates the forms and mechanisms of aquatic creatures like shark skin structures or the coordinated movement of fishes [152,153], is extensively utilized in the design and manufacturing processes. The scientifically designed structure and motion of the vehicle not only effectively reduce drag but also enhance navigation efficiency.

This section evaluates the current application of CFD in biomimetic drag reduction technologies for underwater vehicles. It provides valuable references and insights for researchers in this domain. Liu et al. [154] reviewed various drag reduction methods, assessing their advantages and limitations, and envisioned future advancements. Similarly, Tian et al. [155] conducted an extensive analysis of biomimetic textured surfaces, discussing their morphology, mechanisms, and manufacturing processes, and identified key challenges and future research avenues.

Liu et al. [156] categorized three main types of biomimetic surfaces, elucidating biologically inspired drag reduction and underlining unresolved issues and prospective research areas. Utilizing CFD, several studies have achieved significant strides in underwater biomimetic drag reduction. Malazi et al. [157], for instance, achieved a notable 25% reduction in resistance at a Reynolds number of 1,600,000 using a 3D squid model. He et al. [158], inspired by flounder, developed a biologically inspired flounder two-tier structural surface (BFTSS) that minimized resistance with a synergistic effect, suggesting wide-ranging applications in reducing energy consumption.

**Figure 12:**
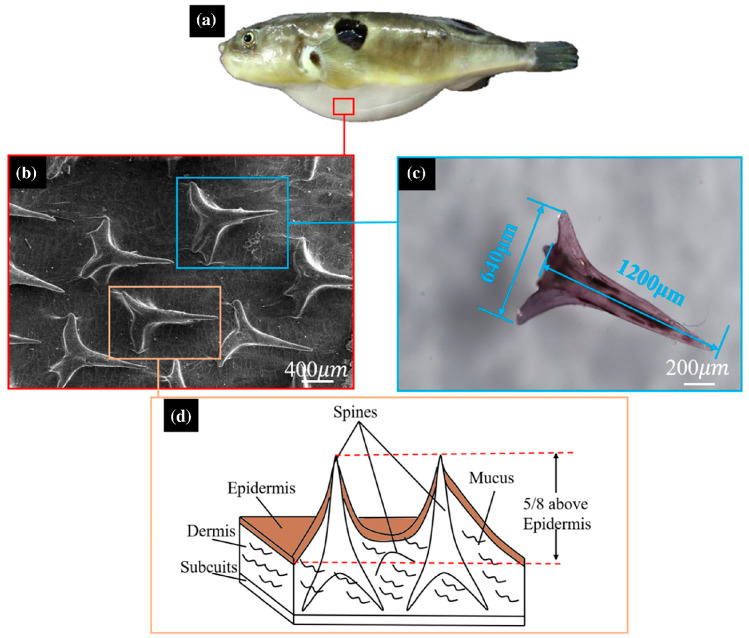
Pufferfish spine structure and SEM images obtained using dried pufferfish skin: (**a**) pufferfish; (**b**) SEM of abdomen spines; (**c**) single-spine structure parameters; and (**d**) spinal structure beneath the epidermis. Reproduced with permission from [160].

Research by Shi et al. [159] and Tian et al. [160] explored the impact of pufferfish spine-inspired structures (as shown in Figure 12) on small AUV hulls, attaining up to a 10.6% reduction in resistance. Extending this concept, Feng’s team [161] ingeniously created a drag-reducing surface on a copper substrate, incorporating cone-shaped protrusions and an elastic layer, inspired by both the spines and skin of pufferfish, achieving significant drag reduction as well as enhanced adhesion and stability.

In the field of underwater drag reduction, the application of biomimetics demonstrates significant diversity and innovation. For instance, Ren et al. [162], influenced by dolphins’ ridge skin and mosquitoes’ mouthparts, demonstrated that underwater drag could potentially be reduced by 89.49% under optimal conditions, thus enhancing operational efficiency. Rostamzadeh-Renani et al. [163] and Natarajan et al. [164] used CFD to study vortex generators inspired by nature, noting resistance reduction in submarines during linear, yaw, and pitch motions.

Xu and his team [165,166], influenced by Sharksuckers, developed an AUV underwater dynamic recovery system, which identified strategies for drag reduction and facilitated more efficient AUV docking. Monfared Mosghani et al. [167], drawing on bony fish scales, designed a ctenoid-shaped microstructure, achieving a 20% average reduction in total resistance under turbulence.

Yuasa et al. [168], taking cues from seal whiskers, investigated how lateral undulations on a cylinder can modify fluid flow vibration frequencies, reducing oscillatory forces. Lu et al. [169] designed a novel AUV, resembling a shark in shape and featuring a water jet pump propeller and sound-absorbing material, to reduce noise. An automated optimization platform was developed for this AUV, leading to a 9.1% reduction in drag and improvements in displacement and energy consumption.

**Figure 13:**
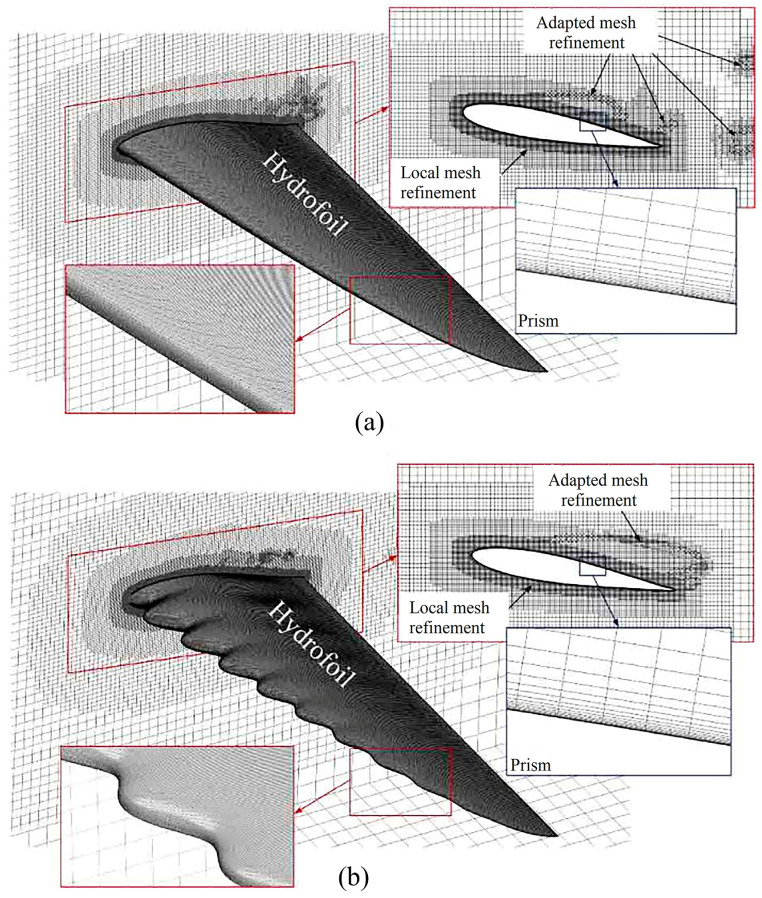
Grid distribution around (**a**) the sinusoidal hydrofoil and (**b**) the biomimetic hydrofoil. Reproduced with permission from [171].

Shukla et al. [170], focusing on FSI, analyzed NACA0012 hydrofoils with surface undulations, drawing inspiration from fish. They identified two vortex modes and variations in thrust. Li et al. [171] used numerical simulations to study cavitation flow fields, inspired by humpback whale fins, and proposed a biomimetic foil with leading-edge bumps, offering insights into enhancing fluid dynamics. The grids of the sinusoidal and biomimetic hydrofoils are depicted in Figure 13.

Similarly, Fish et al. [172] analyzed humpback whale flipper morphology, finding that rounded leading-edge bumps could alter water flow, enhance lift, and reduce drag. Kant and Bhattacharyya [173] explored a dual-bulge hydrofoil design, inspired by humpback whale tubercles, and noted significant lift coefficient changes compared with standard hydrofoils. Chrismianto et al. [174] also conducted CFD analysis on biomimetic humpback whale fin models, achieving up to a 35.13% reduction in total resistance. These studies highlight the potential for these technologies in marine engineering systems.

**Figure 14:**
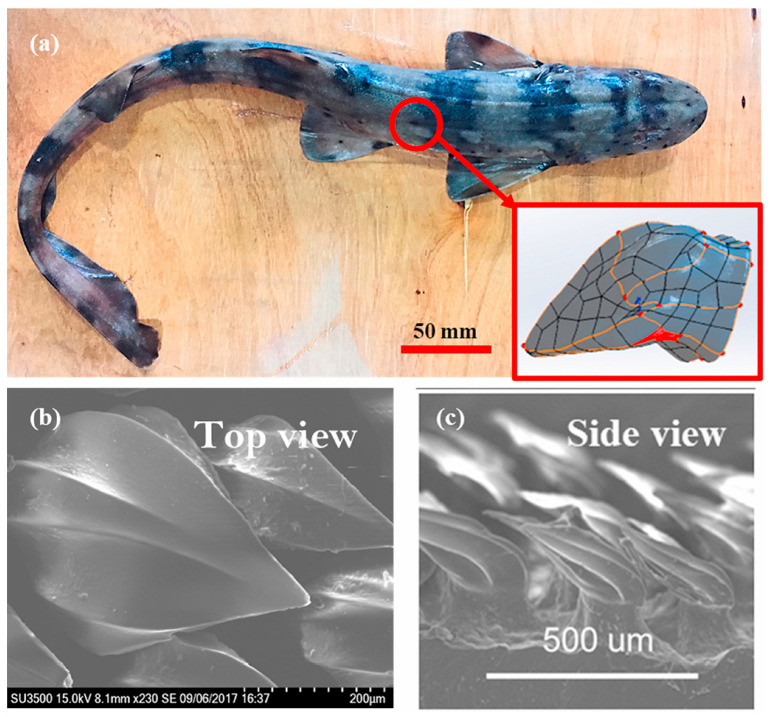
(**a**) Actual shark skin samples. (**b**,**c**) SEM images of dermal denticles on shark skin. Reproduced with permission from [175].

Additionally, Mawignon et al. [175] developed a new numerical optimization method for arranging and orienting 3D rectangular cubic ribs. They discovered that ribs, particularly those perpendicular to the flow, significantly reduce drag. Their study, illustrated in Figure 14, showcases actual shark skin samples and SEM images, highlighting the importance of shark scale structure in enhancing swimming speeds and its potential in maritime applications.

Wu et al. [176] explored aquatic fish scales and proposed a 3D biomimetic model. Using COMSOL Multiphysics for simulations, they demonstrated how these scales can reduce drag by up to 8.40% compared with smooth surfaces at certain water speeds.

Similarly, inspired by hammerhead sharks, Ma et al. [177] designed a head model to study its drag reduction effects. They created a biomimetic robot fish body and used Ansys Mosaic technologies to simulate fluid dynamics, finding improved performance in steady flow fields compared with traditional underwater vehicles.

Furthering the exploration of hammerhead shark-inspired designs, Yan et al. [178] developed a biomimetic fairing for an innovative amphibious robot. Their CFD analysis and experimental studies confirmed significant drag reduction, offering vital insights for amphibious robot navigation design.

Tang’s team, inspired by orca skin, created a unique biomimetic non-smooth surface, leading to a total drag reduction of 11.31%. Their numerical simulations showed the surface’s effectiveness in thickening the turbulent boundary layer and reducing surface friction, presenting substantial potential for engineering applications [179].

These diverse studies collectively provide valuable insights into the design of more efficient underwater vehicles, demonstrating the vast potential of biomimetic approaches in marine engineering.

## 4. Applications of Biomimetic Noise Reduction

Noise reduction is a critical challenge in the development of underwater vehicles, leading to a growing focus on biomimetic approaches for effective solutions. These biomimetic approaches involve drawing inspiration from nature to optimize the structural designs of underwater vehicles. Examples include emulating shark skin’s drag-reduction properties, adopting the tubercle design of humpback whale flippers for flow control, and utilizing the leading-edge serrated structures of owl wings for noise mitigation.

This section focuses on the use of CFD in biomimetic approaches to reduce noise in underwater vehicles, offering a new perspective on flow noise control. Smith and Rigby [180] conducted a comprehensive review of underwater noise sources in marine vessels and evaluated various noise reduction techniques, including biomimetic leading-edge and trailing-edge techniques.

**Figure 15:**
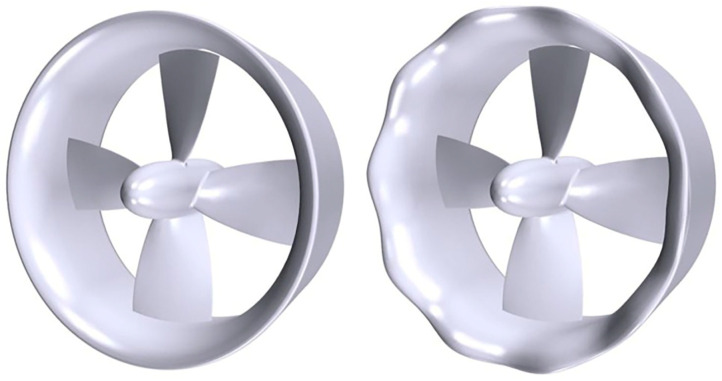
Biomimetic ducted propeller geometry. Reproduced from [181]. CC BY 4.0.

Stark and his team [181,182] innovatively applied tubercles from humpback whale flippers in designing a benchmark ducted propeller. Utilizing Improved Delayed Detached Eddy Simulation and the Ffowcs Williams–Hawkings acoustic analogy, they discovered that these tubercles help in reducing noise by interfering with turbulent structures in the propeller wake, thus accelerating the decay in turbulence and vortex-induced noise. Figure 15 displays the biomimetic ducted propeller’s geometry. Additionally, they adapted these tubercle structures for the propeller blades’ leading edges to mitigate cavitation noise [183].

Trailing-edge serrated structures are widely used in fluid dynamics optimization. Their application extends beyond underwater vehicles, and is even more prevalent in the field of aviation [184,185], particularly for reducing aircraft wake noise. Such designs play a significant role in reducing noise pollution around airports. These structures enhance turbulence within the boundary layer, thereby disrupting and dispersing vortices. This disruption leads to reduced pressure fluctuations and sound wave radiation as the fluid flows over surfaces. In underwater vehicles, serrated ducts effectively alter the wake structure, reducing turbulent kinetic energy and radiated noise.

**Figure 16:**
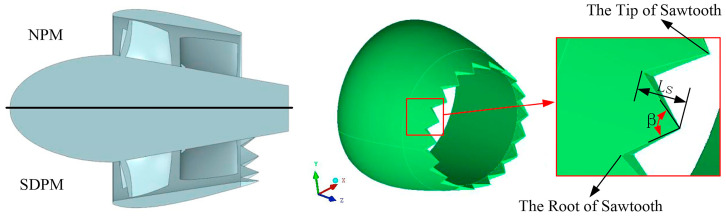
Biomimetic trailing-edge serrated duct. Reproduced with permission from [186].

Qin et al. [186] developed a novel noise reduction technique for pump jet propulsors using a biomimetic trailing-edge serrated duct, as shown in Figure 16. Despite a slight loss in hydraulic efficiency, this design significantly lowers noise across a broad frequency range.

**Figure 17:**
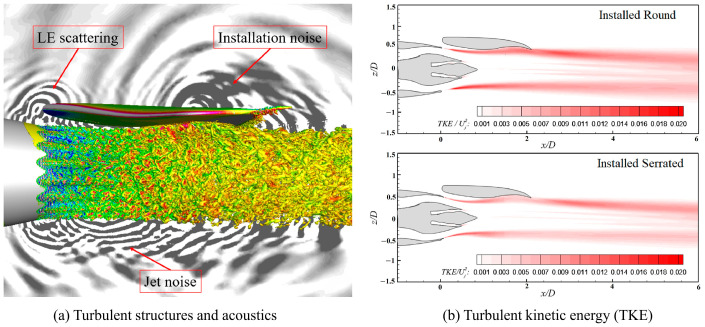
Jet turbulent flows around the wing. Reproduced with permission from [189].

Similarly, aircraft engine exhaust systems benefit from serrated designs, which weaken wake vortices and reduce thermal radiation and acoustic emissions, crucial for stealth capabilities [187,188]. Figure 17 illustrates the efficacy of these serrated structures in aircraft engine exhaust systems, with Figure 17a showing the jet flow and acoustic field, and Figure 17b comparing turbulent kinetic energy between nozzles with and without the biomimetic design, confirming the effectiveness of the serrated design in energy reduction.

**Figure 18:**
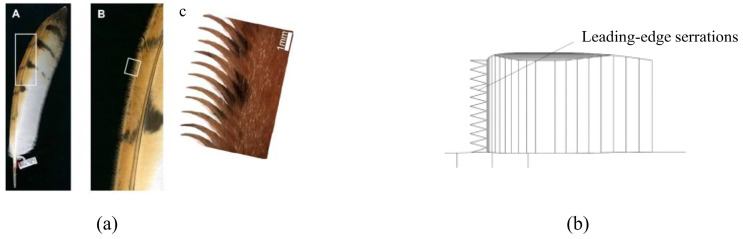
The structures of leading-edge serrations in both owl wings and the sail hull. (**a**) Leading-edge serrations on owl wings. (**b**) Leading-edge serrations on the sail hull of the SUBOFF model. Reproduced from [190]. CC BY 4.0.

Leading-edge serrated structures in underwater vehicles are pivotal in reducing flow noise. Liu et al. [190] investigated the hydrodynamic noise suppression mechanism by integrating leading-edge serrated structures into the SUBOFF model’s sail shell, using both numerical simulations and experimental testing. Figure 18 shows the structures of leading-edge serrations in both owl wings and the sail hull. Their research indicated that this structure induces counter-rotating vortex pairs, disrupts horseshoe vortices, and delays the formation of tail vortices. The findings revealed that with certain leading-edge serrated structural parameters, a hydrodynamic noise reduction of at least 6 dB is achievable within the frequency range of 10 Hz to 2000 Hz, offering a novel approach to designing underwater vehicles with low hydrodynamic noise.

**Figure 19:**
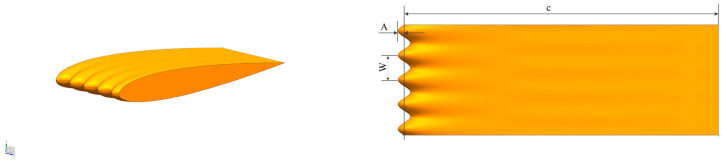
Geometry of the hydrofoil with a wave-shaped leading edge. Reproduced with permission from [191].

Hydrofoils are a crucial part of underwater vehicles. Li et al. [191] conducted a numerical simulation study on the impact of a wave-shaped leading edge on a hydrofoil’s flow structure and noise, as shown in Figure 19. The wave-shaped leading edge not only reduced fluctuations in lift and drag coefficients but also altered the flow field, effectively reducing or even eliminating tonal noise from the hydrofoil without significantly affecting noise directivity. Moreover, they found that at higher inflow speeds, this design is more effective in suppressing lift fluctuations and reducing noise.

**Figure 20:**
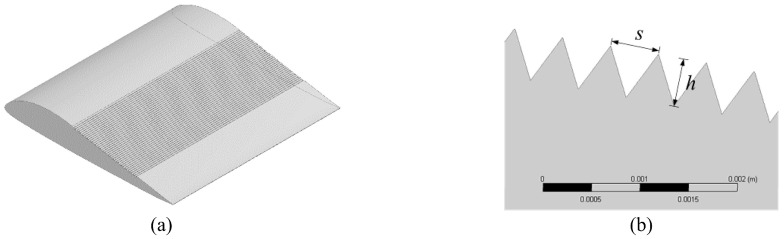
Three-dimensional hydrofoil partially covered with micro-grooves: (**a**) overall model and (**b**) details of the microgrooves. Reproduced from [192]. CC BY 4.0.

Dang et al. [192] achieved further advancements in noise reduction technologies for underwater vehicles by developing a transverse micro-groove surface, inspired by the texture of shark skin. This innovative surface, as depicted in Figure 20, was specifically designed to reduce the hydrodynamic noise of hydrofoils. Their approach involved numerical simulations using LES and Ffowcs Williams–Hawkings equations, complemented by experimental data. The micro-groove surface achieved a significant noise reduction, with a maximum of up to 7.28 dB. This reduction was due to secondary vortices within the micro-grooves that disrupt the turbulence process and reduce the intensity of turbulence bursts.

Superhydrophobic surfaces, products of biomimicry, imitate and design surfaces in nature with superior wetting characteristics. By emulating these unique surface structures and chemical properties found in nature, researchers have developed artificial surfaces with superhydrophobic properties useful for applications such as fluid flow control.

Niu et al. [38] used slip boundary conditions in their simulations to examine the flow patterns on superhydrophobic surfaces at a macro scale. Their research uncovered that these surfaces could substantially reduce hydrodynamic noise, especially at higher frequencies. This reduction was verified with experimental studies, underscoring the effectiveness of superhydrophobic surfaces in noise mitigation.

In subsequent research, they applied superhydrophobic surfaces to control flow-induced noise in underwater cylindrical shells. They compared the flow state and noise levels between a standard cylinder and a superhydrophobic cylinder under high Reynolds number conditions. The results showed that superhydrophobic surfaces could delay flow separation, control wake-shedding vortex size, and significantly reduce flow-induced noise by managing vortex shedding and reducing fluctuation pressure. These surfaces also modified radiation directivity at various frequencies, providing fresh perspectives for underwater vehicle noise control [193].

From the analysis presented above, it is clear that biomimetic noise reduction technology holds substantial potential and value in managing noise for underwater vehicles. By replicating specific biological and physical features found in nature, this approach can significantly improve the acoustic stealth performance of underwater vehicles. Furthermore, these studies also provide invaluable experience and serve as a reference for future research and practical applications.

## 5. Discussion

CFD, when integrated with biomimicry, offers expansive prospects for the development of underwater vehicles, despite facing potential challenges and limitations. The following discourse explores these limitations and sheds light on promising future applications.

### 5.1. Challenges and Prospects in Biomimetic Propulsion

Biomimetic propulsion technology is an instrumental area of research in the design of underwater vehicles. Numerous marine creatures, including fishes, batoids, dolphins, and squids, utilize unique propulsion systems that significantly inspire designers. While essential in simulating such propulsion systems, the use of CFD faces distinct challenges.

Firstly, many biomimetic propulsion methods use complex FSI, making accurate simulation with CFD models highly challenging. For instance, the movements of dolphins or the jetting techniques of squids involve dynamic boundary conditions coupled with structural nonlinear responses, presenting an arduous challenge for CFD models.

Secondly, most marine organisms generate propulsion through rhythmic oscillations of one or multiple body segments. Simulating such unconventional and irregular motion paths requires high computational accuracy and incurs significant computational costs in CFD.

Furthermore, although biomimetic propulsion design has unique advantages, replicating natural designs often involves high complexity. For example, mimicking the dynamic swinging of fish fins or dolphin bodies requires intricate mechanical design, precise control systems, and effective integration with CFD.

Moreover, biological propulsion methods, while exceptionally efficient, often rely on specific speeds and environments. Biomimetic propulsion systems may not match the efficiency of conventional propulsion systems under varying working conditions and speed ranges.

Despite significant challenges, the application of CFD to biomimetic propulsion holds promising prospects, driven by advancements in computational techniques, materials science, Artificial Intelligence (AI) integration, and interdisciplinary collaborations.

Emerging computational methods are set to revolutionize CFD applications in biomimetic propulsion. Advanced algorithms and multi-scale models are being developed to more accurately simulate the complex FSI inherent in biological systems. These innovations promise greater fidelity in replicating the nuanced dynamics of marine organisms at various scales. Additionally, the integration of AI and Machine Learning (ML) offers transformative potential in design optimization, with AI-driven algorithms capable of analyzing complex datasets to uncover new propulsion principles and autonomously refine designs, thereby accelerating innovation.

Material science plays a crucial role in mimicking the sophisticated structures of biomimetic propulsion systems. Current research is focusing on developing new materials, like bio-inspired polymers, to emulate the flexibility and strength of biological tissues, which is vital for the mechanical feasibility and environmental sustainability of biomimetic systems.

The future of biomimetic propulsion also hinges on interdisciplinary collaboration, combining insights from biology, fluid dynamics, and robotics. This collaborative approach is essential for understanding and replicating the efficiency and adaptability of biological propulsion mechanisms.

Scaling from small-scale laboratory models to full-scale operational systems presents significant prospects. It requires innovative engineering solutions and careful consideration of compatibility with existing underwater vehicle technologies. Additionally, the environmental impact and sustainability of these systems are paramount, with biomimetic propulsion offering a greener alternative to conventional methods, potentially reducing energy consumption and noise pollution in marine environments.

Economically, the field of biomimetic propulsion is at a crucial juncture. Its commercial viability, encompassing research and development costs, manufacturing, and maintenance, requires thorough evaluation. The potential markets, spanning military applications to oceanographic research, offer diverse opportunities for the application of these technologies.

In summary, although confronted with considerable challenges, the field of biomimetic propulsion in underwater vehicles is poised for significant advancements. With ongoing technological progress and a multidisciplinary approach, the application of CFD in this domain holds vast potential, promising a new era of efficient, sustainable, and innovative underwater propulsion systems.

### 5.2. Challenges and Prospects in Biomimetic Drag Reduction and Noise Reduction

In the field of biomimetic drag reduction and noise reduction, researchers often focus on replicating features from the same creature, such as shark skin, to study these aspects in underwater vehicles. Although these two issues are typically studied separately, they are fundamentally related. This discussion aims to explore the challenges, limitations, and future prospects of biomimetic drag reduction and noise reduction in underwater vehicles.

Firstly, CFD shows great potential in researching biomimetic drag reduction and noise suppression for underwater vehicles. However, various challenges and limitations exist in practical applications. One primary challenge is the limited understanding of nature; current research often replicates common traits observed in certain creatures, but simulation results may not align with actual observations due to difficulties in simulating complex natural factors like fluid boundary conditions, physiological properties, and environmental influences on animal behavior.

Secondly, the accuracy of CFD simulations heavily relies on the chosen turbulence model. Most of these models are empirical or semi-empirical and may not fully capture the complexities of flows. This limitation can lead to inaccuracies in predicting the effects of biomimetic drag reduction and noise reduction.

Moreover, using CFD simulation in marine environments requires substantial computational resources and time, especially for intricate 3D models. These constraints hinder the swift design and optimization process of underwater vehicles. Additionally, the precision and reliability of CFD simulations are often restricted, as current methodologies may require simplifications in complex computations, potentially compromising accuracy.

Furthermore, most existing CFD models concentrate primarily on single physical fields, like flow field simulation, making it challenging to address issues related to noise generation and propagation, which involve the interplay of sound and the flow fields. The primary sources of noise in underwater vehicles are diverse, including propeller, mechanical, and hydrodynamic noise, necessitating precise CFD and acoustic calculation models for effective biomimetic noise reduction design.

In addition, designing biomimetic noise reduction systems typically requires extensive biological studies, which are costly and need a significant amount of experimental and observational data. Furthermore, these models require high accuracy in describing fluids, structures, acoustics, and their interactions, demanding high computational capabilities and multidisciplinary optimization within CFD.

The application of CFD in biomimetic drag reduction and noise suppression for underwater vehicles, though currently facing challenges, holds considerable promise for future advancements. A primary focus is on refining CFD methodologies to more accurately simulate the intricate FSI, integrating realistic environmental conditions and physiological properties of marine organisms, thereby bridging the gap between theoretical models and real-world observations.

Interdisciplinary research is another key area. A synergistic approach, combining insights from biology, fluid dynamics, and material science, is essential. This collaboration will deepen our understanding of biological mechanisms and facilitate their replication in biomimetic designs. Addressing computational efficiency is also crucial, as current CFD models require substantial resources. Future research should focus on developing algorithms that optimize computational load without sacrificing accuracy, including the use of parallel computing and reduced-order models.

A notable gap in current CFD models is the integration of high-precision acoustic calculations. Developing comprehensive models that can simulate both fluid and sound fields will greatly enhance our capability to understand and reduce hydrodynamic noise. This requires sophisticated modeling that can predict noise generation and its propagation in underwater environments.

The potential of AI and ML in biomimetic design is immense. These technologies could revolutionize the field by optimizing drag reduction and noise suppression features, using data-driven algorithms to rapidly identify efficient designs.

Furthermore, integrating extensive experimental and observational studies is vital for validating and refining computational models. These studies provide empirical data that are crucial for enhancing model accuracy and underscore the need for developing cost-effective methods for biological studies to enable more comprehensive data collection.

Lastly, a holistic approach to biomimetic design optimization is needed, integrating fluid dynamics, structural mechanics, acoustics, material science, and control engineering within a multidisciplinary design optimization framework. This approach is key to enhancing the overall performance of underwater vehicles.

In conclusion, while the current application of CFD in biomimetic drag reduction and noise suppression faces several challenges, the field is on the brink of transformative advancements. The evolution of computational technologies, combined with a strong emphasis on interdisciplinary collaboration, is set to significantly enhance CFD’s role in optimizing underwater vehicle design, leading to more efficient, sustainable, and innovative marine propulsion systems.

## 6. Conclusions

In recent years, as the importance of ocean exploration has become increasingly recognized, the role of underwater vehicles has become crucial. Thanks to rapid advancements in computational capabilities and improvements in numerical simulation methods, CFD technology now offers reliable numerical solutions for the complex physical systems of underwater vehicles.

Compared with traditional design strategies, the integration of CFD technology with multi-physics fields and multi-disciplinary optimization significantly shortens product development cycles and reduces research and development costs. This approach has led to more efficient and cost-effective design processes for underwater vehicles.

Biomimetics, as a cross-disciplinary research field, has played a significant role in the design and development of underwater vehicles. Researchers are increasingly drawing inspiration from the morphology, structure, and functions of biological species to design and manufacture underwater vehicles with novel functions. This biomimetic approach enhances the flexibility, maneuverability, and adaptability of these vehicles to complex marine environments.

The integration of CFD technology and biomimetics provides not only a reliable and effective method for understanding the mechanisms of biomimetic propulsion, drag reduction, and noise reduction in underwater vehicles but also opens up new possibilities for future oceanic development and scientific research. This synergy between CFD and biomimetics is paving the way for innovative solutions in marine exploration and technology.

## Figures and Tables

**Table 2:** Overview of CFD methods and numerical techniques for fluid dynamics simulations.

Category	Method	Description	Typical Applications
Numerical Methods	FVM	Solves equations using discrete control volumes.	Versatile, used in many commercial CFD software.
	FEM	Uses mesh of elements, effective for complex geometries.	Structural analysis, fluid dynamics.
	BEM	Focuses on boundaries, reduces 3D problems to 2D.	Potential and external flow problems.
Turbulence Models	RANS	Averages Navier–Stokes over time for steady and turbulent flows.	Industrial applications, steady-state flows.
	URANS	Extends RANS to unsteady flows.	Vortex shedding, transient flows.
	DES	Hybrid of RANS and LES for flows with separated regions.	Aerospace, automotive industries, and underwater vehicles.
	LES	Resolves large-scale turbulent structures, models smaller scales.	Detailed turbulence research, complex flows.
	DNS	Simulates all turbulent flow scales without modeling.	Fundamental turbulence research.
Mesh-free Methods	SPH	Particle-based method for simulating free-surface flows.	Astrophysics, engineering, and environmental modeling.
Statistical Methods	LBM	Simulates fluid flow using particle distribution functions.	Complex, multiphase flows.
Vortical Flow Methods	Vortex	Focuses on capturing vortical structures in incompressible flows.	Aerodynamics, turbulent flow simulations.

## Data Availability

Not applicable.
